# Forecasting the spatial and seasonal dynamic of *Aedes albopictus* oviposition activity in Albania and Balkan countries

**DOI:** 10.1371/journal.pntd.0006236

**Published:** 2018-02-12

**Authors:** Clément Tisseuil, Enkelejda Velo, Silvia Bino, Perparim Kadriaj, Kujtim Mersini, Ada Shukullari, Artan Simaku, Elton Rogozi, Beniamino Caputo, Els Ducheyne, Alessandra della Torre, Paul Reiter, Marius Gilbert

**Affiliations:** 1 Spatial Epidemiology Lab. Université Libre de Bruxelles, Brussels, Belgium; 2 Control of Infectious Diseases Department, Institute of Public Health, Tirana, Albania; 3 National Veterinary Epidemiology Unit, Food Safety and Veterinary Institute, Tirana, Albania; 4 Department of Biology, Faculty of Natural Sciences, University of Tirana, Tirana, Albania; 5 Department of Public Health and Infectious Diseases, University of Rome “Sapienza”, Rome, Italy; 6 European Economic Interest Group—European Agro-Environmental Health Geographic Information Systems, Zoersel, Belgium; 7 Insects and Infectious Disease Unit, Institute Pasteur, Paris, France; Faculty of Science, Mahidol University, THAILAND

## Abstract

The increasing spread of the Asian tiger mosquito, *Aedes albopictus*, in Europe and US raises public health concern due to the species competence to transmit several exotic human arboviruses, among which dengue, chikungunya and Zika, and urges the development of suitable modeling approach to forecast the spatial and temporal distribution of the mosquito. Here we developed a dynamical species distribution modeling approach forecasting *Ae*. *albopictus* eggs abundance at high spatial (0.01 degree WGS84) and temporal (weekly) resolution over 10 Balkan countries, using temperature times series of Modis data products and altitude as input predictors. The model was satisfactorily calibrated and validated over Albania based observed eggs abundance data weekly monitored during three years. For a given week of the year, eggs abundance was mainly predicted by the number of eggs and the mean temperature recorded in the preceding weeks. That is, results are in agreement with the biological cycle of the mosquito, reflecting the effect temperature on eggs spawning, maturation and hatching. The model, seeded by initial egg values derived from a second model, was then used to forecast the spatial and temporal distribution of eggs abundance over the selected Balkan countries, weekly in 2011, 2012 and 2013. The present study is a baseline to develop an easy-handling forecasting model able to provide information useful for promoting active surveillance and possibly prevention of *Ae*. *albopictus* colonization in presently non-infested areas in the Balkans as well as in other temperate regions.

## Author summary

The Asian tiger mosquito *Aedes albopictus*, originating from Asia, in the last decade has spread in many regions in Europe and US. Beside the nuisance problem causing to the citizens during the day, this species has raised public health concern, due to its strict association with humans and anthropic habitats, its expanding distribution and its capacities to transmit several human arboviruses. We developed a spatio-temporal model of *Ae*. *albopictus* dynamics that helps to understand the biology and the ecology of the species in relation to environmental factors, and to inform efficient control strategies. Here we developed a dynamical species distribution modeling approach at high spatial (over the Balkans) and temporal resolution (weekly scale), enabling to link oviposition activities and climatic conditions across different time periods to forecast the potential future oviposition activities of *Ae*. *albopictus* in unknown locations or identify target areas and periods of highest activities. Extrapolating *Ae*. *albopictus* abundance over the Balkan region may help to identify habitat suitability where the species has never been reported so far. The temperature-related predictors remain the most determinant predictors among all candidate predictors e.g land cover and rainfall. The model provides useful information for promoting active surveillance on *Ae*. *albopictus* and assessing the risk of exotic arbovirus transmission in temperate regions.

## Introduction

In the last decade, the increasing spread of the Asian tiger mosquito, *Aedes albopictus*, in Europe and US has raised public health concern, as the species is involved in the transmission of several human arboviruses among which dengue, chikungunya and Zika [1,2,3,4]. The tiger mosquito is arrived in Europe in the seventies, probably through cargo transportation from China [5]. The first record of this species in Europe was reported from Albania in 1979, although it is quite possible that the species was already present in mid-1970s, at least two decades before the species was first detected in Italy in 1991[5]. Nowadays, *Ae*. *albopictus* is widespread and commonly found in Albania, even in tiny isolated villages and sites in high altitude including beech forest up to 1200m. In other European countries, Italy reported *Ae*. *albopictus* up to 600m altitude[6] and the species was found in Switzerland, France or Spain and widely distributed in Balkan countries such as Croatia, Montenegro and Serbia [(http://ecdc.europa.eu/en/healthtopics/vectors/vector-maps/Pages/VBORNET_maps.aspx) [7]

To mitigate the potential impact of the mosquito in transmitting human diseases, efforts were made to better understand the biology and the ecology of the species. The role of the environmental factors in the spread and temporal dynamics of *Ae*. *albopictus* has been investigated in both laboratory and field work conditions [8]. Temperature is shown to be a crucial driver for *Ae*. *albopictus* activity at different levels, from adult abundance [9] and oviposition activity [10], to eggs incubation [11] and eggs hatching [12,10,11]. More specifically, laboratory studies showed to which extent the strength of thermal conditions, their starting period and duration, could impact spawning and embryogenesis [11]. Some other environmental factors related to land cover, have been shown to be statistically associated with high habitat suitability for *Ae*. *albopictus* larval breeding site, through the landscape structure [13].

To date, several studies have focused on modeling the spatial distribution of *Ae*. *albopictus* at different spatial scales, from global [14], to continental for Europe, [15,16] or country and regional scales e.g., for Japan, [17] for Northern Italy [18,19]. However, as most classical species distribution modeling approaches, those studies provide a static picture of *Ae*. *albopictus* activity, by statistically relating the occurrence [14,15] or eggs abundance [20] of the species to some environmental spatial factors for some fixed period of the year. By contrast, a few studies have either accounted for spatio-temporal variables [21,22,23] or developed dynamical models of *Ae*. *albopictus* biological activity [24], enabling to link oviposition activities and climatic conditions across different time periods [25]. The use of dynamical model provides a pragmatic solution for public health policies to forecast the potential future oviposition activities of *Ae*. *albopictus* or identify target areas and periods of highest activities. This might be used, in return, to develop efficient scenarios to mitigate the impact of the tiger mosquito in the spread of several human diseases among which dengue, chikungunya and Zika.

Hereby, we present a three-year study of the abundance of *Ae*. *albopictus* eggs at different altitudes on Dajti mountain in central Albania. We developed a novel dynamical species distribution modeling approach at high spatial and temporal resolution. Our major goal is to develop a statistical forecasting model as simple as possible in terms of implementation for non-statistician users, with relatively low computer-time calculation and flexibility for extrapolating projected results to varying spatial and temporal scales. Our approach contrast with the many species distribution models published previously by allowing spatially and temporally explicit predictions. The three main objectives are: i) calibrating and validating a forecasting approach based on independent data to assess the ability of the model to project the potential future oviposition of *Ae*. *albopictus* in unknown locations; ii) ensuring that the strength of environmental drivers on mosquito spawning, as fitted by our model at large spatial and time scale, is consistent with literature review; iii) extrapolating our forecasting approach and project the spatio-temporal oviposition activity of *Ae*. *albopictus* over the Balkans between 2009 and 2012 at high spatial (1km^2^ resolution approximately) and temporal resolution (weekly scale).

## Methods

### Forecasting model framework

The forecasting modeling framework was based on five major steps summarized hereafter. First, the entomological and environmental data were collected and stored into a spatial-temporal database for data analysis. Second, a calibration step aimed at selecting the best combination of predictors and statistical models that best fit the observed spatio-temporal patterns of eggs abundance. Third, the ability of the forecasting model to project unforeseen future events in new locations was tested on independent validation dataset. Four, the forecasting model projections were extended to the entire Balkan countries (Albania, Montenegro, Macedonia, Serbia, Kosovo, Greece, Croatia, Slovenia, Bulgaria and Rumania) at high spatial (one km) and high temporal (week) resolution between 2009 and 2013. The overall modeling framework was built upon Generalized linear models (GLM; [26]) to facilitate the ecological understanding of model behavior while minimizing computer-time calculations and favoring the replication of model to similar ecological systems.

### Dataset

#### Entomological survey

Oviposition activity of *Ae*. *albopictus* was monitored in 26 sites across Albania by ovitraps, i.e. black cylindrical vessels (9 cm high, 11 cm in diameter with an overflow hole at 7 cm from the base) filled with ~300 ml tap-water with no attractants, and internally lined with heavy-weight seed germination paper [27]. No specific permissions were required for these sites/activities. Landowners gave permission to conduct the studies on their properties. The field studies did not involve endangered or protected species.

**Fig 1:**
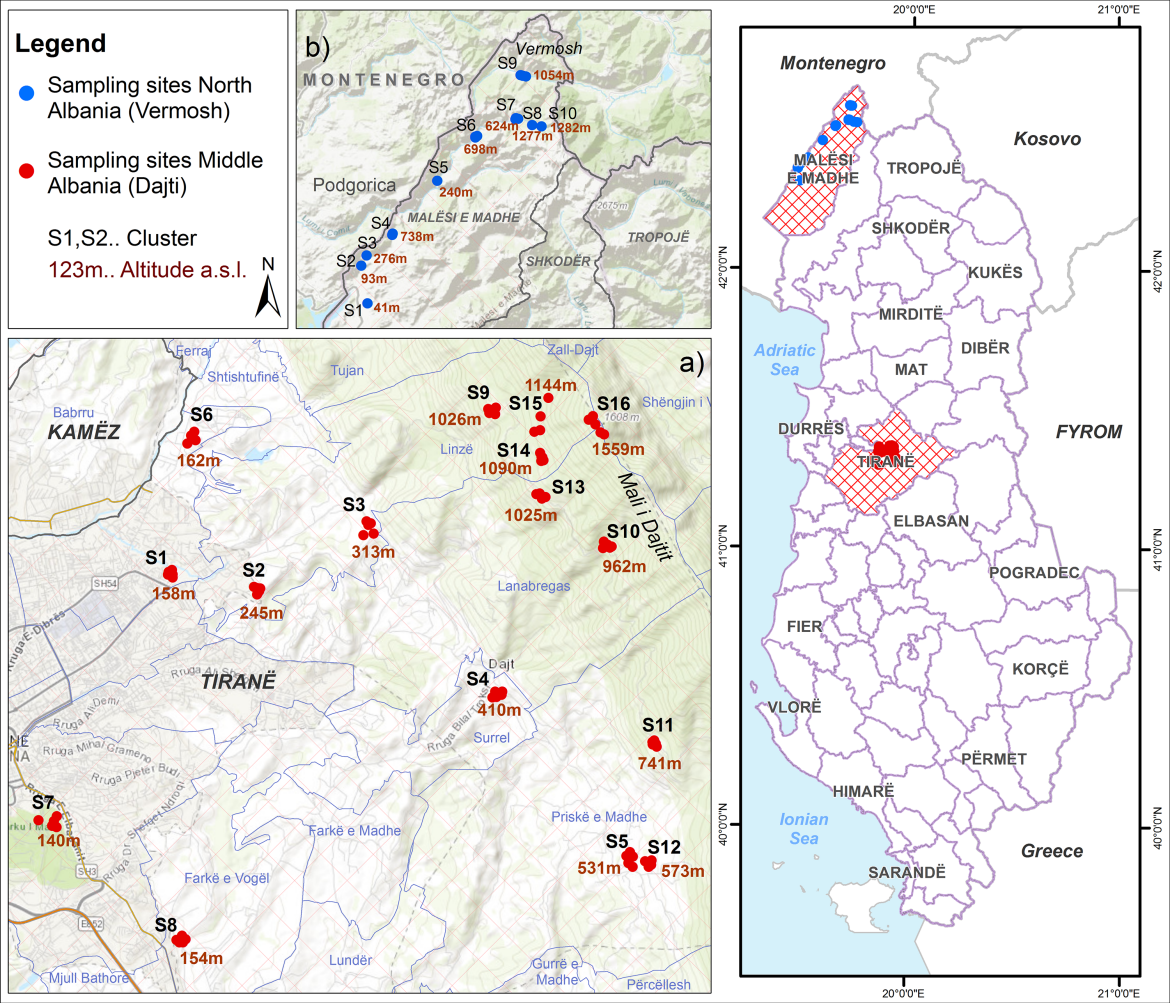
Location of the sampling sites. a) 16 sites used for model calibration are located in Middle Albania (Tirana-Dajti mount); b) 10 sites used for model validation are located in North Albania (Malesia e Madhe–Vermosh). Information sources are open source information from: http://asig.gov.al/ https://landsatlook.usgs.gov/viewer.html.

A first set of 16 sites located in the center of Albania (Tirana-Dajti mount, 19 55'51.2"Eo, 41 21'34.5"No, across a 154–1559 meter altitude gradient, (Fig 1A) was used for model calibration.

Monitoring started in April 2011 and continued on a weekly basis until December 2013, by 5 ovitraps/site (i.e.80 ovitraps/week). A second set of 10 sites located in the Northern part of Albania (Malesia e Madhe-Vermosh 19 41'26"E, 42 35'22"N, along an altitude gradient ranging from 41 to 1282 meters, (Fig 1B) was used for model validation.

In the latter 10 sites, monitoring was carried out in August-September 2012 every two weeks by 5 ovitraps/site and the number of eggs/week was calculated by dividing the total number of eggs collected over the two weeks by two.

Eggs laied on germination paper were counted and identified to species level based on their color, size, shape and surface sculpting [28].

#### Environmental data

Climate and landscape data expected to influence *Ae*. *albopictus* ecological dynamic were used to derive relevant predictors in the model [21, 22, 23]. The mean land surface temperature (LST) were extracted from Modis data, by averaging night and daily LST data, then rescaling data to the weekly resolution using spline interpolation to match the sampling unit of the study. Altitude data were based on the Shuttle Radar Topography Mission database (SRTM; [29]). For each site, the dominant class of Corine land-cover 1km resolution raster [30] was extracted among: artificial surface, agriculture, forest, wetlands or water body.

### Forecasting model structure

The spatial and temporal sampling unit of study was defined at the site and week scale, respectively i.e. the forecasting model aimed to predict the total abundance of *Ae*. *albopictus* eggs/site/week.

The structure of the forecasting model was made of three components; namely the 'Core', 'Init', and 'Max' components. While the Core component is the key feature, making the direct link between the spatial and temporal variability of environmental predictors and the abundance of *Ae*. *albopictus* eggs/site/week, the ‘Init’ and ‘Max’ component defines the initial and maximal condition values inside which the Core modeling component was allowed to run.

#### The Core model

Ten predictors expected to influence eggs abundance were used in the models. We aimed to develop a parsimonious model using a relatively limited number of variables with a plausible biological causal relationship underlying the predictions. The variables included: i) three biological predictors referring to the respective eggs abundance recorded at each three weeks before the sampling; ii) four climate predictors including the mean temperature recorded at the sampling week and at each of the past three weeks of sampling, in order to take into account the role of climate variables during the larval development in affecting *A*. *albopictus* adult abundance and survival [23]; iii) two geophysical predictors related to Corine land-cover and altitude; iv) one seasonal predictor denoting the week of the year the sample was recorded.

Considering eggs abundance as count data, Poisson (hereafter referred as 'poisson') or negative binomial (hereafter referred as 'nb') errors distribution families were assumed in the modeling setup. In addition, simple GLMs (hereafter referred as 'glm') as well as more complex types of model enable to account for the presence of large number of zeros in the database (> 50%) were also tested; namely hurdle (hereafter referred as 'hurdle') and zero-inflated (hereafter referred as 'zeroinf') models [31]. It is worth to note that zero-inflated models are based on a zero-inflated probability distribution i.e. a distribution that allows for frequent zero-valued observations. So, the zero inflated model fit simultaneously two separate regression models. On one hand a logistic or probit model that predicts the probability of being a non-zero count, and on the other hand a model that predicts the size of that count. In total, six models derived from the combination between the three types of model and the two distribution families were compared to each other; i.e. glm-poisson, glm-negbin, hurdle-poisson, hurdle-negbin, zeroinf-poisson, zeroinf-negbin.

#### The Init and Max models

The Init modeling component defined initial condition values to allow the Core component to initialize projections from any week of the year and any location, and to populate the core model with these values to generate projections. This allows projections to be made in any location where the predictor covariate data is available. Similarly, the Max modeling component defined maximal condition values at each step of the forecasting process, preventing the Core model to project some excessive or unreliable eggs abundance values above those maximal condition values.

Based on the calibration dataset, the mean and maximum weekly eggs abundance over the three years of sampling was calculated at each site. The Init and Max models were setup individually, by regressing the mean (for the Init model) and the maximum (for the Init model) weekly eggs abundance against the mean altitude and the week of the year (as a second polynomial degree). GLMs models with Poisson and negative binomial distribution families were tested.

#### Models selection

The procedure used to select the best combination of predictors and statistical models was inspired from [32]. For each sub-model (i.e. Core, Init and Max model), each statistical model was first built using the full set of potential predictors, hereafter referred as 'full model'. Then, the 'full' model that best fulfilled the following five criteria was selected: 1) normality of residuals; 2) homogeneity of residuals; 3) absence of strong spatial and temporal autocorrelation patterns in the residuals as quantified by the experimental semi-variogram; 4) dispersion parameter as close as possible of value 1; 5) Akaike information criterion as small as possible (AIC). Finally, a stepwise procedure was applied to the best 'full' model to derive the most parsimonious 'final' model i.e. the model which maximizes the model goodness-of-fit based on AIC criteria while minimizing the number of predictors. All predictors were transformed to normality and scaled to zero mean and unique variance. All models were calibrated using the calibration dataset.

#### Dynamical feature

The dynamical feature of the forecasting model was designed to refine the accuracy of projections while integrating meaningful ecological information related to the biological cycle of *Ae*. *albopictus*. Technically, this was implemented in 4 steps by: i) initializing the Core model at a given week of the year (Wt) using the Init Model by estimating starting egg counts; ii) Output from the Init Model were used to derive the egg count predictor variables to be included in the Core Model; iii) Core model projections were then calculated while paying attention that they do not exceed Max model projections, otherwise they were given the corresponding Max model projection value; iv) Core model outputs at time Wt were used as egg count predictors at time Wt-1 to derive Core model projections at time Wt+1; v) The model was run iteratively until the ending week of the year, by repeating step iii) and iv).

### Forecasting model calibration and validation

The forecasting model was calibrated using the calibration dataset while validated using the validation dataset. Both dataset were assumed to be spatially independent, so that the validation step provided a suitable assessment of the forecasting model ability to extrapolate projections to higher spatial extents. Since the validation data were only available in weeks 32, 34, 36 and 38 for year 2012, the goodness-of-fit assessment was made for this period only.

Model goodness-of-fit and uncertainty was evaluated throughout bootstrap approach under 100 iterations. For each iteration, 70% of the calibration dataset was randomly sampled (with replacement) to calibrate a single forecasting model. The projections derived from the 100 forecasting models were then assembled to calculate the 10^th^, 50^th^ and 90^th^ percentiles projections values.

The model goodness-of-fit was quantified by comparing the projected values with the validation observational values using root mean square error (RMSE) and Spearman rank correlation coefficients index. The robustness of the forecasting model was discussed in terms of weekly variability and by initializing the model at different weeks of the year, ranging from one to five weeks before the first recording week.

### Forecasting model extrapolation to the Balkans

Once validated, the forecasting model was extrapolated to Albania and its surroundings countries (Montenegro, Macedonia, Serbia, Kosovo, Greece, Croatia, Slovenia, Bulgaria, Rumania) at 0.01 degree spatial resolution (WGS84 coordinate reference system), and at a weekly interval from 2009 to 2013. Model projections are provided as supplementary information data S1 Dataset.

The forecasting model assumed stationarity, so that the relationships fitted between the abundance of eggs and its predictors was supposed to remain stable beyond the spatio-temporal extent of model calibration.

All statistical analyses were performed under the R software environment (version 3.1.1) using packages pscl [33], visreg [34], dplyr [35], and ggplot2 [36].

## Results

### Entomological observations

The entomological survey revealed the presence of *Ae*. *albopictus* even in very tiny and isolated sites up to the altitude 1,200 m a.s.l. in Middle Albania (Dajti mount) and up to 1,054 m a.s.l. in North Albania (Vermosh). The highest species abundance (i.e. 1028 eggs/ovitrap in average w 28 (second week of July), 2012) was observed in park and gardens in urban, suburban and rural areas below 550 m a.s.l and high abundance was also observed up to 700 m a.s.l. in both the North and the Central transects (i.e. 377 eggs/ovitrap in average w31, 2013). While at altitudes <160 m oviposition continued from May to early December (weeks 19–49, with peaks in August and September), the activity declined with at higher altitudes and was observed only from end of June to end of September (w25-w38) at >760 m a.s.l..

Although *Ae*. *albopictus* eggs represented >97% of mosquito eggs found, eggs of other mosquito species were also found in ovitraps. *Ochlerotatus geniculatus* eggs were found in almost all stations along the transect in Dajti mount from May (w19) to beginning of September (w36) at altitudes <140 m a.s.l. and from beginning of June (w24) to end of August (w35) at higher altitudes. *Anopheles sp*. eggs were found in ovitrap located in the 333 m a.s.l. site in Dajti mount (w25, 2012). Presence of *Anopheles plumbeus* adults was observed at 1257m a.s.l., end of June 2012 (w25). *Culex pipiens* egg rafts were found in the 962 m a.s.l. site in Dajti mount in June-July 2012 (w25-28).

### Model selection

The first model selection step consisted in identifying the most suitable statistical models for the Core, Init and Max modeling components, independently. For the Core modeling component, zero-inflated negative binomial model displayed the best goodness-of-fit results, with particularly low AIC, RMSE and dispersion parameters values (zeroinf-nb; Table 1).

**Table 1:** Statistical model selection for the Core, Init and Max modeling components, based on based on Akaike information criterion (AIC), Spearman correlation coefficient (COR), root mean square error (RMSE) and dispersion parameter (DISP).

Statistical model	Core Model				Init Model				Max Model			
	AIC	COR	RMSE	DISP	AIC	COR.	RMSE	DISP.	AIC	COR.	RMSE	DISP.
Poisson model (glm-pois)	31174.	0.76	125	103.7	89731	0.82	97.6	72.12	150582	0.81	164.5	124.42
Negative binomial model (glm-nb)	-	0.76	29274	7.9	10446	0.83	126.9	2.91	11627	0.81	231.7	2.61
Hurdle Poisson model (hurdle-pois)	190935	0.77	115	19.1	-	-	-	-	-	-	-	-
Hurdle negative binomial model (hurdle-nb)	22227	0.79	404	0.76	-	-	-	-	-	-	-	-
Zero-inflated poisson model (zeroinf-pois)	190935	0.79	115	19.32	-	-	-	-	-	-	-	-
Zero-inflated negative binomial model (zeroinf-nb)	22207	0.79	376	0.74	-	-	-	-	-	-	-	-

‘-’ Statistical models that have not been tested as candidate models.

**Fig 2:**
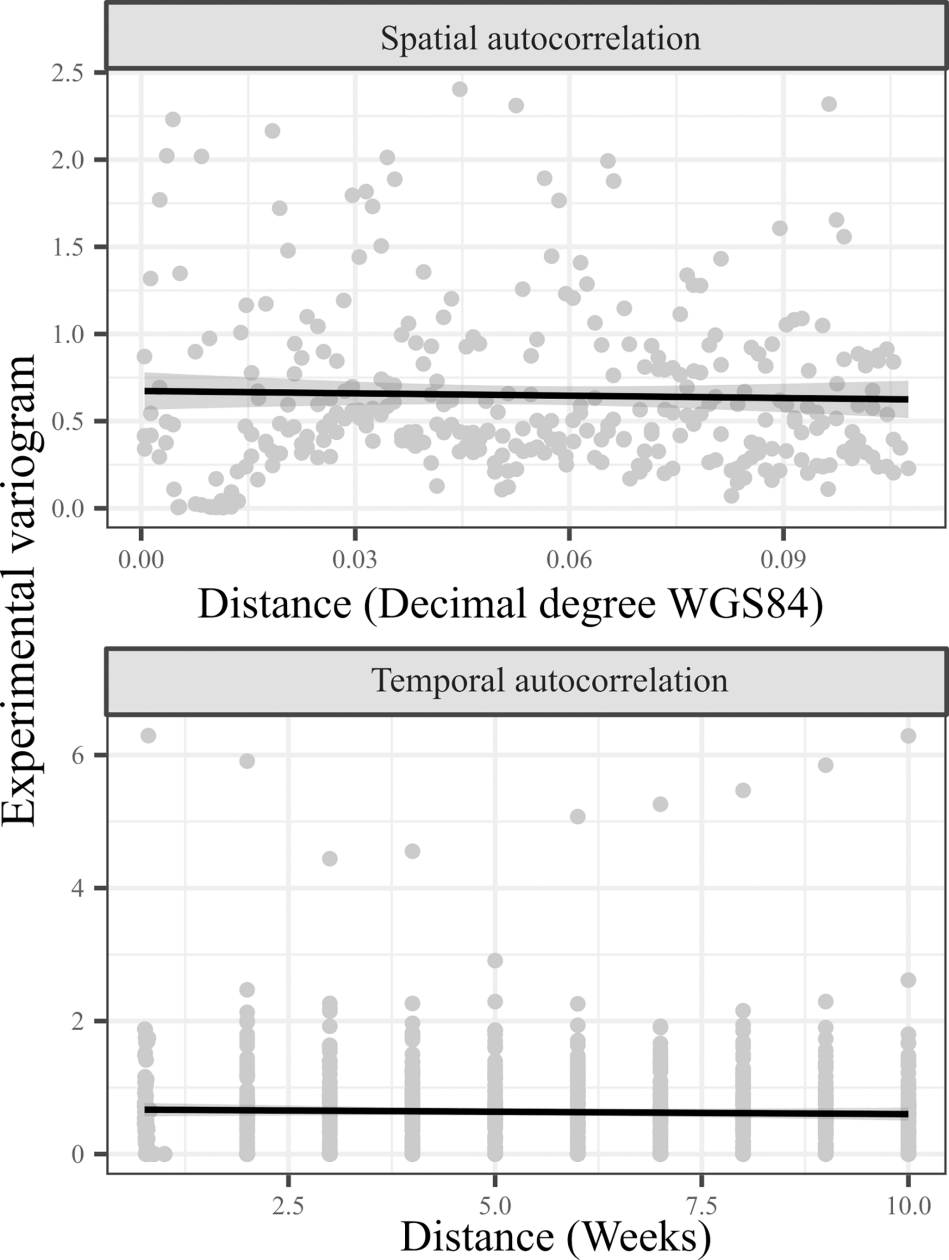
Spatial and temporal experimental semi-variograms calculated from the final Core model residuals, highlighting low autocorrelation in model residuals (the black line indicated the smoothed trend).

The best statistical model was selected based on the following criteria: normality, homogeneity and absence of strong spatial and temporal autocorrelation patterns in the residuals (Fig 2), minimizing dispersion, RMSE and AIC parameter values. All models were built upon the calibration dataset.

In regards with the Init and Max modeling component, best results were obtained using GLM statistical model with negative binomial distribution family (glm-nb; Table 1).

In addition, the analysis of Core model results revealed a satisfying normality, homogeneity as well as an absence of strong spatial and temporal autocorrelation patterns in residuals (Fig 2).

Final Core, Init and Max modeling components were derived using AIC backward stepwise variable elimination and results are shown in Table 2.

**Table 2:** Parameters, standard deviation and significance for the best Core (zero inflated negative binomial model; zeroinf-nb), Init (GLM negative binomial model; glm-nb) and Max models (GLM negative binomial model; glm-nb).

	Core model (zeroinf-nb)						Init model (glm-nb)			Max model (glm-nb)		
	*count model*			*zero model*								
* *	Coef.	Sd.	Sig	Coef.	Sd.	Sig	Coef.	Sd.	Sig	Coef.	Sd.	Sig
Intercept	4.24	0.04	***	-0.22	0.09	*	-15.68	-1.32	***	-15.91	-1.39	***
Egg count _Week-1_	0.25	0.03	***	-1.59	0.21	***	-	-	-	-	-	-
Egg count _Week-2_	0.12	0.03	***	-0.65	0.15	***	-	-	-	-	-	-
Egg count _Week-3_	0.08	0.02	**	-0.4	0.12	**	-	-	-	-	-	-
Temperature _Week-1_	0.34	0.04	***	-0.87	0.16	***	-	-	-	-	-	-
Altitude	-0.35	0.05	***	-0.01	0.00	***	-0.01	0.00	***	-0.01	0.00	***
Week of the year	-	-	-	-	-	-	1.37	-0.08	***	1.40	-0.08	***
Week of the year^2^	-	-	-	-	-	-	-0.02	0.00	***	-0.02	0.00	***

*, **, ***, Predictors significance at the alpha level of 0.05, 0.01 and 0.001 respectively.

‘-’Predictors which are not included in the modeling setup.

### Model behavior

**Fig 3:**
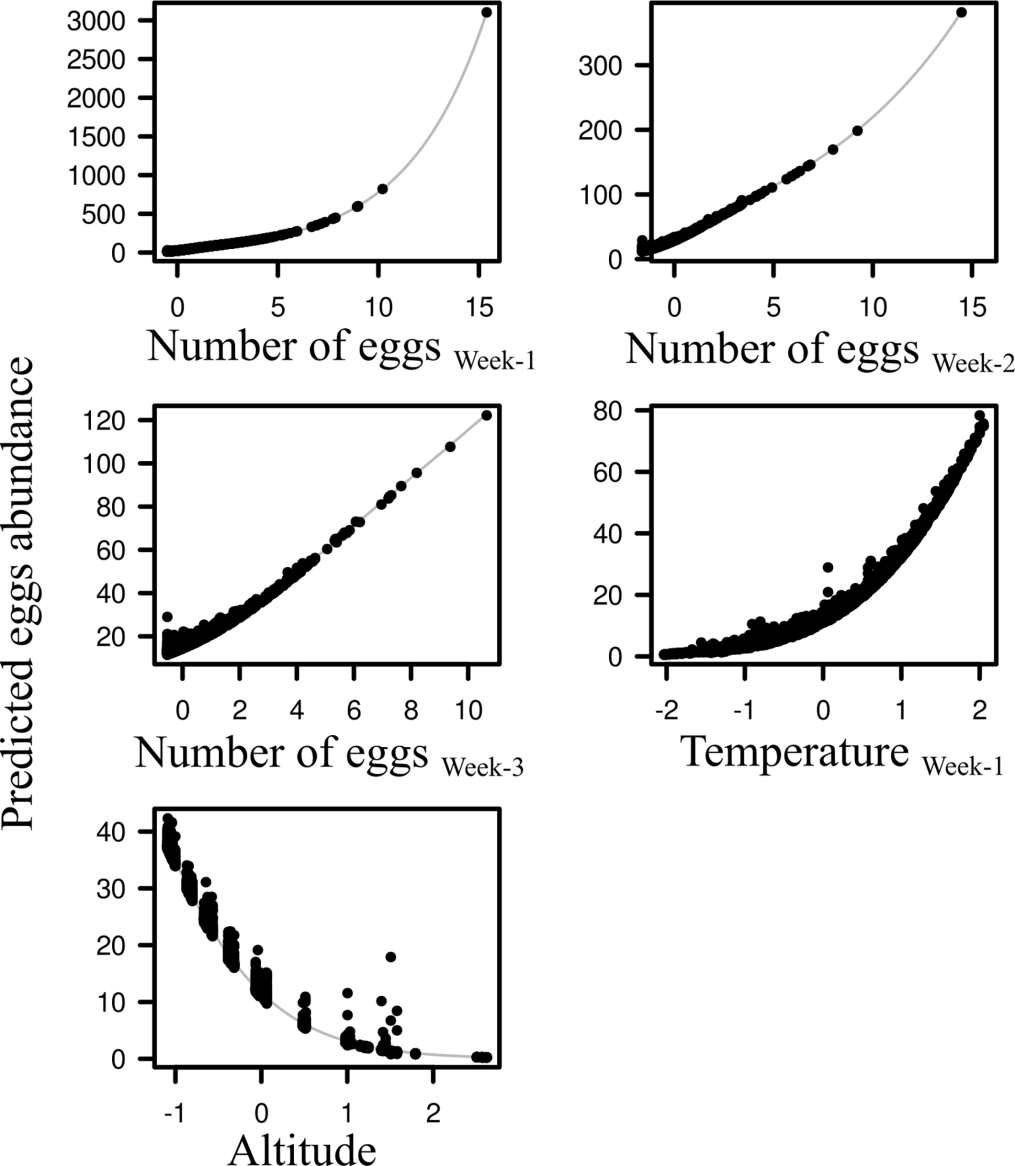
Conditional effects of selected predictors (x-axis) on the egg abundances (y-axis) in the the final Core zero-inflated negative binomial model.

Based on the final Core model results, the main relationships between conditional effect of predictors and eggs abundance was shown in Fig 3. One can note that the temperature in the current week, the lagged temperature in the weeks -2 and -3, and the land cover variables were taken out of the significant predictor variables by the backward stepwise procedure.

The influence of egg abundance in the previous weeks decreased with time, with the strongest effect of egg abundance at week-1, and the lowest but yet significant effect for egg abundance at week-2 and week-3 (Table 2 and Fig 3). Temperature measured by the MODIS LST signal in the previous week had positive influence on eggs abundance, while altitude displayed a negative effect. Neither the land cover nor the seasonal predictors were selected in the final model. This suggests the seasonal eggs abundance variability was satisfactorily captured by climatic and previous week egg abundance predictors.

### Model validation

**Fig 4:**
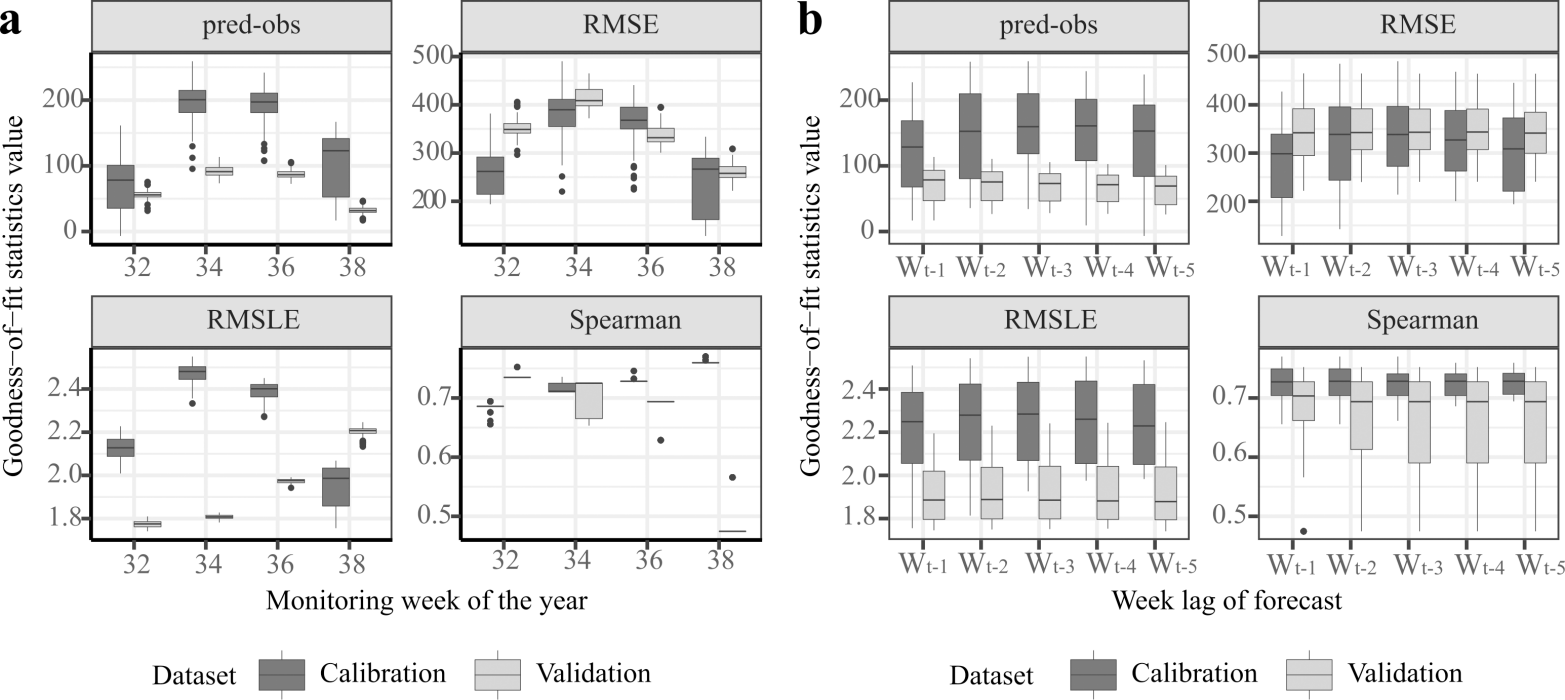
Goodness-of-fit assessment for the forecasting model applied to the validation and calibration sites, based on 100 bootstrap model projections. a) according to the monitoring week of the year and b) according to the week of model initialization.

The forecasting model accuracy was evaluated using calibration and validation sites for year 2012 during the summer period (weeks 32–38). Results from the 100 bootstrap model outputs are summarized in Fig 4.

Goodness-of-fit criteria included the residuals (i.e. the difference between the predicted and observed abundance), root mean squared error (RMSE) and Spearman correlation values. Since the validation data were only available for weeks 32, 34, 36 and 38 in year 2012, the overall goodness-of-fit assessment was performed for this period only.

Model accuracy was assessed in terms of weekly variability (Fig 4A) as well as by initializing the model at different weeks of the year, ranging from one to five weeks before the first recording week (Fig 4B).

Globally, goodness-of-fit metrics were relatively comparable between calibration and validation dataset, although validation metrics values were moderately lower than the calibration ones e.g. residuals _calibration_ ≈ 140.37 *vs* residuals _validation_ ≈ 67.14, RMSE _calibration_ ≈ 310.41 *vs* RMSE _validation_ ≈ 342.15, Spearman _calibration_ ≈ 0.72 *vs* Spearman _validation_ ≈ 0.66 (Fig 4). Those results indicated the relatively good ability of the forecasting model to extrapolate out of its spatial range of calibration.

The seasonal variability in model performances were relatively low and stable across time, although the model displayed noticeably better performances in weeks 32 and 38 than in weeks 34 and 36 (Fig 4A). Similarly, the variability in model performances due to the different initialization weeks of the year was relatively low as well (Fig 4B). This highlighted the overall good forecasting capacity of the model.

### Model projections

**Fig 5:**
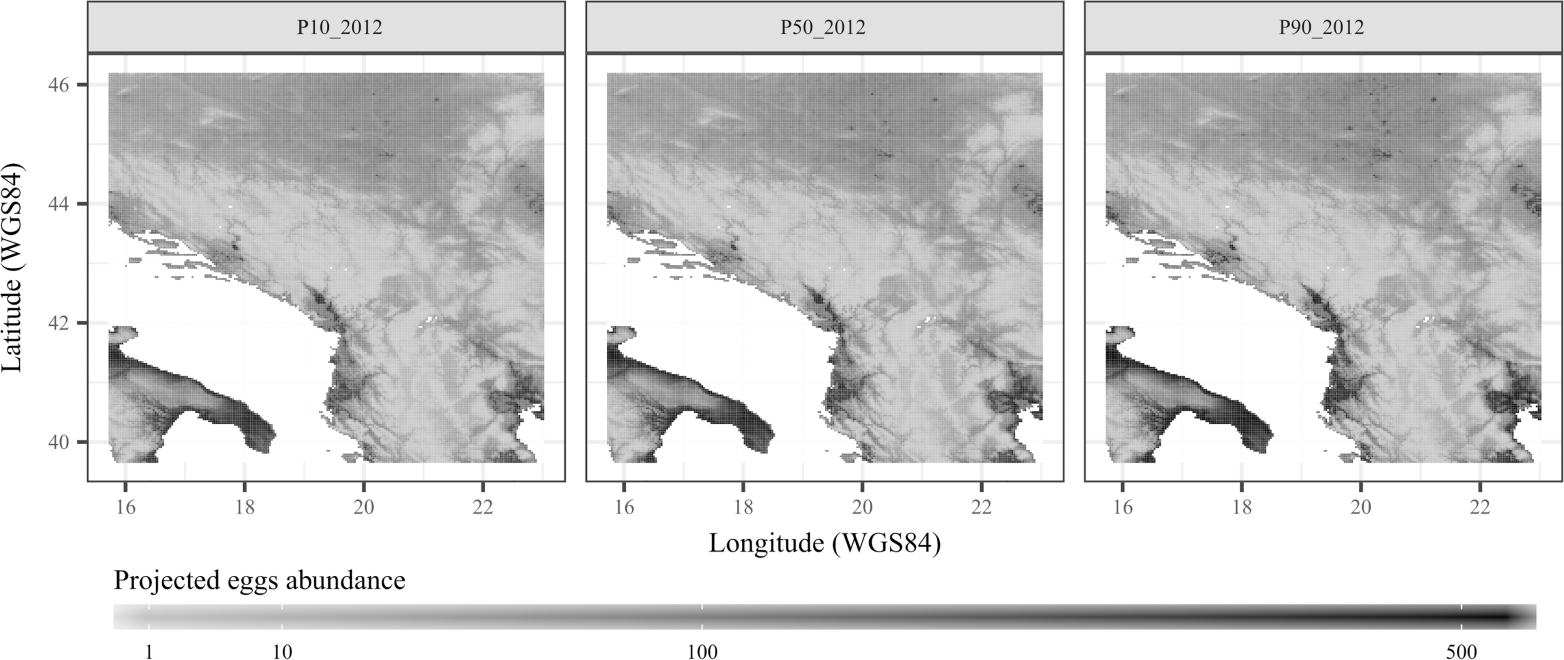
Annual mean percentiles of 10% (p10), 50% (p50) and 90% (p90) of *Aedes albopictus* eggs abundance projected by the forecasting model over Balkan countries in year 2012. The darker the color the more projected abundance by the model.

**Fig 6:**
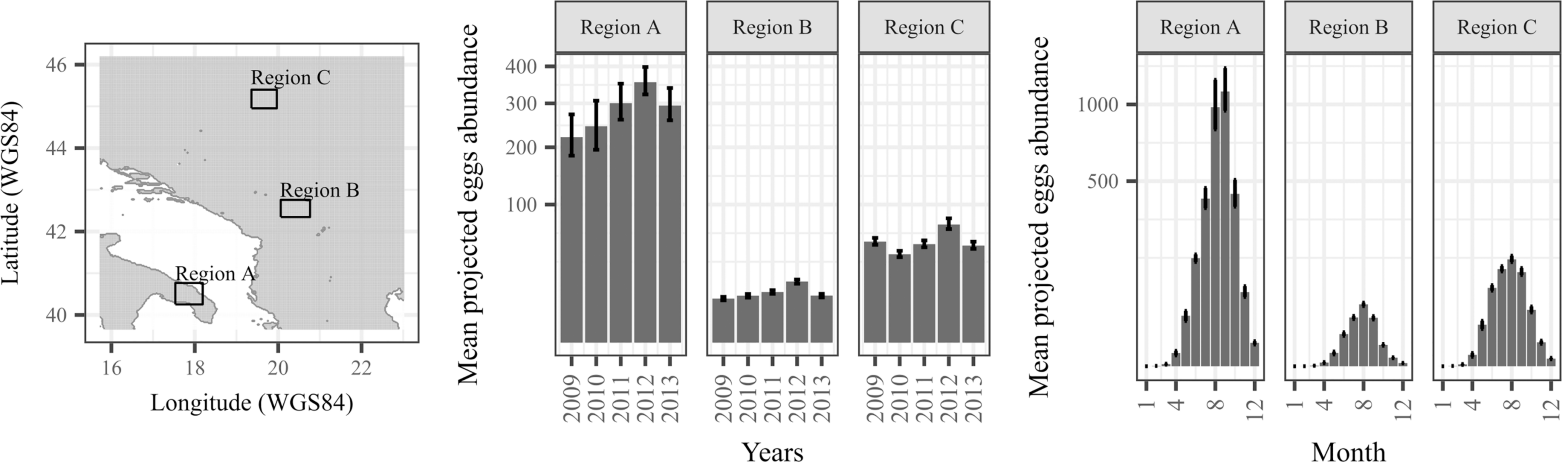
Mean annual and monthly *Aedes albopictus* eggs abundance projected by the forecasting model over the period 2009–2013 for the three regions located in the southern (region A), middle (region B) and northern part (region C) of Balkan countries. The central statistics (percentile p50) and the associated error bars (percentiles p10 and p90) of each barplot were calculated from 100 bootstrap models projections, averaged over the three regions of interest.

The forecasting model was successfully applied to high spatial (0.01 WGS84 decimal degrees) and temporal (weekly) resolutions over the Balkans for the period 2009–2013. Summary statistics (percentiles 10, 50 and 90) were derived from the 100 bootstrap model runs to assess the mean trend and uncertainty in the projected eggs abundance spatiotemporal patterns (Figs 5 and 6).

Results were averaged to derive a spatial distribution maps in eggs abundance for each year. The spatial patterns being relatively similar between years, results for year 2012 are shown to illustrate the main spatial trends (Fig 5). Globally, results displayed strong spatial heterogeneity, the southern coastal regions displaying higher eggs abundance than in northern and central regions. Resulting maps for percentiles 10, 50 and 90 were very similar to each other. This indicated that the spatial uncertainty related to model error was relatively low.

The results were analyzed annually and seasonally for three different regions located in the southern (region A), middle (region B) and northern (region C) parts of Balkans (Fig 6).

Although the three regions displayed different results in terms of magnitude, each region displayed a strong similar seasonal signal. The projected annual peaks in eggs abundance generally occurred between the summer months of August and September i.e. approximately from weeks 32 to 38. Importantly, the annual and seasonal uncertainty in projections was relatively low since the mean percentiles 10 and 90 values were closed to the median value.

## Discussion

Our results provide confidence in the ability of the here proposed forecasting model to project the spatial and seasonal oviposition activity of *Ae*. *albopictus* over Albania and its neighboring countries. Firstly, the goodness-of-fit indicators from the calibration and validation step were satisfying. In regards with the Core modeling component, the use of zero-inflated model was shown to outperform other GLMs models. In particular, zero inflated models have the interest to account both for the skewed distribution (generally best modeled using negative binomial distribution family) and for the high proportion of zero values in eggs samples. While similar spatial modeling studies of *Ae*. *albopictus* generally apply negative binomial distribution family to their model, they do not take into account high proportion of zero values [23,22,21]. Secondly, the spatio-temporal projections validated on the Northern sites of Albania, provides confidence in the ability of model to extrapolate the spatio-temporal dynamics of eggs abundance over the other Balkan countries. Some noise may however, have been added in this validation by the fact that the sampling interval was different in the training and validation data, but given the fairly good goodness-of-fit metrics, we believe that this effect was low.

It is noteworthy that the geographical extrapolation exercise remains questionable over longer geographical distance, as far as the model has not validated using observational data from the neighboring Balkan countries, which was not feasible in the frame of the present work, but may become feasible in the near future as more information on the *Ae*. *albopictus* abundance in the region are becoming available [37]. One should note, that in many cases, the validation of spatial models is internal through the use of cross-validation, so fact of training and validating the model in entirely different areas goes beyond the state-of-the-art encountered in many species distribution models applied to disease vectors. However, despite this limitation, extrapolating *Ae*. *albopictus* abundance over the Balkan region may help to identify suitable environmental niche where the species has never been reported so far.

From a biological point of view, our results are in line with the known literature [23,22,21] and provide further insights on the predominant predictors of *Ae*. *albopictus* oviposition activity. The key predictor was related to antecedent oviposition activities from the previous week. That is, the abundance of eggs for a given week is likely to generate a proportional abundance of adults which will spawn, in return, a proportional abundance of eggs for the next-coming week. Temperature from the previous week was the second most important predictors of eggs abundance, presumably through the influence of temperature on adults spawning. Thus, these results are consistent with the known biological activity of *Ae*. *albopictus*, [38] which in temperate areas have developed the ability to induce photoperiodic egg diapause, allowing overwintering and further assisting its establishment in more northerly latitudes. [11] Altitude and seasonal information related to the week of sampling was of minor influence on eggs activity. It is worth to note that temperature-related predictors are seemingly correlated with both altitude and seasonality, thus spatio-temporal times series of temperatures are likely to capture some seasonal and altitudinal signal. Altitude can be a proxy for temperature, but may is also a proxy for slope or insolation, which may impact oviposition activity. Land cover was not shown to be a determinant factor influencing oviposition activity, however this effect might be significant in some particular areas not covered by our sampling design e.g. proximity to water bodies. Some other predictors could have been included to improve the model such as water-related predictors that may strongly influence oviposition activity, [39] in particular the development time from eggs immersion to the adult state. However, the satisfying predictive power of our model confirms that the temperature-related predictors remain the most determinant predictors among all candidate predictors.

The proposed forecasting model provide information useful for promoting active surveillance and possibly prevention of *Ae*. *albopictus* colonization in presently non-infested areas in the Balkans (e.g. Kosovo and Macedonia), as well as in high altitude areas, and could represent a helpful instrument for assessing the actual risk of exotic arbovirus transmission in temperate regions.

## Supporting information

S1 Dataset Spatial and temporal model projections over the Balkans (Albania, Montenegro, Macedonia, Serbia, Kosovo, Greece, Croatia, Slovenia, Bulgaria, Rumania) at 0.01 degree spatial resolution (WGS84 coordinate reference system), and at a weekly interval from 2009 to 2013. (PDF)
