# Supplementary material for: Forecasting the spatial and seasonal dynamic of Aedes albopictus oviposition activity in Albania and Balkan countries
Source: PLoS Negl Trop Dis. 2018 Feb 12;12(2):e0006236. doi: 10.1371/journal.pntd.0006236 (PMC5825170; doi:10.1371/journal.pntd.0006236)
Supplement: S1 Dataset — (PDF) [file pntd.0006236.s001.pdf]

Supporting information: Projected eggs abundance data over the Balkans at high spatial and temporal resolution.

Data characteristics

- Seven NetCDF files showing the spatio-temporal projections derived the 100 boostrap models runs
  - forecast.Q5.nc : Quantile 5
  - forecast.Q25.nc : Quantile 25
  - forecast.Q50.nc : Quantile 25
  - forecast.Q75.nc: Quantile 75
  - forecast.Q90.nc: Quantile 95
  - forecast.Q95.nc: Quantile 95
  - forecast.MEAN.nc : Mean
- Grid coordinates :
  - lonlat : points=478805 (731x655)
  - longitude : 15.7211 to 23.0211 by 0.01 degrees_east
  - latitude : 46.1937 to 39.6537 by -0.01 degrees_north
- Vertical coordinates :
  - surface
  - levels=1
- Time coordinate :
  - 247 steps
  - Format: YYYY-MM-DD hh:mm:ss
  - 2009-01-23 00:00:00; 2009-01-30 00:00:00; 2009-02-06 00:00:00; 2009-02-13 00:00:00 2009-02-20 00:00:00; 2009-02-27 00:00:00; 2009-03-06 00:00:00; 2009-03-13 00:00:00; 2009-03-20 00:00:00; 2009-03-27 00:00:00; 2009-04-03 00:00:00; 2009-04-10 00:00:00; 2009-04-17 00:00:00; 2009-04-24 00:00:00; 2009-05-01 00:00:00; 2009-05-08 00:00:00; 2009-05-15 00:00:00; 2009-05-22 00:00:00; 2009-05-29 00:00:00; 2009-06-05 00:00:00; 2009-06-12 00:00:00; 2009-06-19 00:00:00; 2009-06-26 00:00:00; 2009-07-03 00:00:00; 2009-07-10 00:00:00; 2009-07-17 00:00:00; 2009-07-24 00:00:00; 2009-07-31 00:00:00; 2009-08-07 00:00:00; 2009-08-14 00:00:00; 2009-08-21 00:00:00; 2009-08-28 00:00:00; 2009-09-04 00:00:00; 2009-09-11 00:00:00; 2009-09-18 00:00:00; 2009-09-25 00:00:00; 2009-10-02 00:00:00; 2009-10-09 00:00:00; 2009-10-16 00:00:00; 2009-10-23 00:00:00; 2009-10-30 00:00:00; 2009-11-06 00:00:00; 2009-11-13 00:00:00; 2009-11-20 00:00:00; 2009-11-27 00:00:00; 2009-12-04 00:00:00; 2009-12-11 00:00:00; 2009-12-18 00:00:00; 2009-12-25 00:00:00; 2010-01-22 00:00:00; 2010-01-29 00:00:00; 2010-02-05 00:00:00; 2010-02-12 00:00:00; 2010-02-19 00:00:00; 2010-02-26 00:00:00; 2010-03-05 00:00:00; 2010-03-12 00:00:00; 2010-03-19 00:00:00; 2010-03-26 00:00:00; 2010-04-02 00:00:00; 2012-10-29 00:00:00; 2012-11-05 00:00:00; 2012-11-12 00:00:00; 2012-11-19 00:00:00; 2012-11-26 00:00:00; 2012-12-03 00:00:00; 2012-12-10 00:00:00; 2012-12-17 00:00:00;

2012-12-24 00:00:00; 2012-12-31 00:00:00; 2013-01-28 00:00:00; 2013-02-04 00:00:00; 2013-02-11 00:00:00; 2013-02-18 00:00:00; 2013-02-25 00:00:00; 2013-03-04 00:00:00; 2013-03-11 00:00:00; 2013-03-18 00:00:00; 2013-03-25 00:00:00; 2013-04-01 00:00:00; 2013-04-08 00:00:00; 2013-04-15 00:00:00; 2013-04-22 00:00:00; 2013-04-29 00:00:00; 2013-05-06 00:00:00; 2013-05-13 00:00:00; 2013-05-20 00:00:00; 2013-05-27 00:00:00; 2013-06-03 00:00:00; 2013-06-10 00:00:00; 2013-06-17 00:00:00; 2013-06-24 00:00:00; 2013-07-01 00:00:00; 2013-07-08 00:00:00; 2013-07-15 00:00:00; 2013-07-22 00:00:00; 2013-07-29 00:00:00; 2013-08-05 00:00:00; 2013-08-12 00:00:00; 2013-08-19 00:00:00; 2013-08-26 00:00:00; 2013-09-02 00:00:00; 2013-09-09 00:00:00; 2013-09-16 00:00:00; 2013-09-23 00:00:00; 2013-09-30 00:00:00; 2013-10-07 00:00:00; 2013-10-14 00:00:00; 2013-10-21 00:00:00; 2013-10-28 00:00:00; 2013-11-04 00:00:00; 2013-11-11 00:00:00; 2013-11-18 00:00:00; 2013-11-25 00:00:00; 2013-12-02 00:00:00; 2013-12-09 00:00:00; 2013-12-16 00:00:00; 2013-12-23 00:00:00; 2013-12-30 00:00:00
